# Exogenous plant growth regulators alleviate natural high temperature stress after anthesis in wheat (*Triticum aestivum* L.) by sustaining efficient photosynthesis and antioxidant defense responses

**DOI:** 10.3389/fpls.2026.1926064

**Published:** 2026-09-25

**Authors:** Feiyan Ju, Fengtao Zhao, Chunhua Gao, Weilin Kong, Zongxin Li, Haijun Zhao, Kaichang Liu, Ping Liu

**Affiliations:** 1 Institute of Industrial Crops, Shandong Academy of Agricultural Sciences, Jinan, China; 2 State Key Laboratory of Nutrient Use and Management, Key Laboratory of Agro-Environment in Huang-Huai-Hai Plain, Institute of Agricultural Resources and Environment, Shandong Academy of Agricultural Sciences, Jinan, China; 3 Shandong Academy of Agricultural Sciences, Jinan, China

**Keywords:** antioxidant defense, natural high temperature stress, photosynthetic efficiency, plant growth regulator, wheat (*Triticum aestivum* L.)

## Abstract

**Introduction:**

High temperature stress during the grain-filling period severely suppresses plant growth and yield formation in winter wheat. Plant growth regulators including 2,4-epibrassinolide (EBR) and melatonin (MT) have been reported to effectively enhance plant thermotolerance, representing a promising strategy to mitigate extreme high temperature stress and stabilize wheat yield.

**Methods:**

In this study, integrated agronomic, physiological and transcriptomic analyses were conducted to explore the regulatory effects of different exogenous plant growth regulators on thermotolerance and physiological characteristics of winter wheat during the grain-filling stage.

**Results and discussion:**

Exogenous application of these regulators modulated the expression of core photosynthesis-related genes, increased the net photosynthetic rate of flag leaves, enhanced photosynthetic capacity, and alleviated heat-induced damage. Meanwhile, exogenous regulators altered the transcript levels of key antioxidant enzyme-coding genes, thereby boosting antioxidant enzyme activities, reducing the accumulation of reactive oxygen species (ROS) and malondialdehyde (MDA), and relieving membrane lipid peroxidation. Coordinated regulation of photosynthetic and antioxidant metabolic pathways substantially elevates the thermotolerance of winter wheat against natural high temperature stress after anthesis. The Mix treatment (0.05 µmol L^-1^ 2,4-epibrassinolide + 50 µmol L^-1^ melatonin + 15 mmol L^-1^ KH_2_PO_4_) markedly optimized the physiological and biochemical status of heat-stressed wheat, alleviated biomass loss and increased grain yield. Collectively, this study improves the core regulatory mechanisms by which exogenous plant growth regulators mediate thermotolerance in winter wheat during grain filling, and provides new theoretical references and practical cultivation strategies for maintaining wheat yield and grain quality under high temperature stress.

## 1 Introduction

The World Meteorological Organization (World Meteorological Organization (WMO), 2026) reported 2023–2025 as the hottest three-year period on record and forecasted a 1.3-1.9 °C rise in global near-surface mean temperature (pre-industrial baseline: 1850–1900) during 2026-2030, with global temperatures likely staying at record-high levels. Wheat (*Triticum aestivum* L.) is a globally predominant staple crop, covering 216 million hectares and supplying major dietary calories worldwide. As a winter cereal, wheat exhibits a critical growth threshold of 26 °C, rendering it highly susceptible to rising temperatures (Abhinandan et al., 2018; Molero et al., 2023). Intensifying climate change exacerbates field heat stress, disrupting agricultural systems and causing crop yield losses exceeding 50%, which severely endangers global food security (Lamaoui et al., 2018; Gu et al., 2023). Increasing extreme heat events throughout the wheat growing season, particularly during the grain-filling stage, substantially limit crop productivity (Zhu et al., 2022; Yuan et al., 2023; Arif et al., 2025). Therefore, developing advanced cultivation and stress mitigation techniques to improve wheat heat tolerance is essential for sustaining food security under progressive climate warming (Zhang et al., 2024).

Extreme heat impairs wheat through multi-layered regulatory pathways, modifying crop-climate interactions and physiological feedbacks to substantially suppress plant growth and grain yield (García et al., 2015; Cohen et al., 2021). As a cool-season C3 crop, wheat is highly susceptible to heat stress, with the early grain-filling stage being its most temperature-sensitive phase (Liu et al., 2014; Ullah et al., 2022; Jiang et al., 2025). Frequent, intensified heatwaves during this critical period trigger cellular injury and premature senescence while disrupting grain-filling dynamics, thereby restricting biomass accumulation, reducing grain weight, and ultimately degrading wheat yield and quality (Sarto et al., 2017; Riaz et al., 2021; Hafeez et al., 2023). Existing evidence shows that wheat grain filling proceeds optimally at 20-24 °C, and each 1 °C rise in ambient temperature reduces grain yield by approximately 6% (Farooq et al., 2011; Asseng et al., 2015; Mohan et al., 2023). After anthesis, temperatures exceeding 32 °C accelerate senescence, suppress flag leaf photosynthesis, shorten the grain-filling duration, and reduce 1000-grain weight, thereby causing substantial yield losses (Abdelrahman et al., 2020; Abdelhakim et al., 2021). Consistently, high temperature stress after anthesis primarily constrains wheat yield by reducing grain number per spike and individual grain weight (Tao et al., 2015; Girousse et al., 2021). Beyond yield penalties, heat stress remodels a suite of physiological and biochemical processes in wheat. Severe heat disrupts thylakoid architecture and induces lipid peroxidation of chloroplast membranes (Liu and Huang, 2000; Ristic et al., 2007; Djanaguiraman et al., 2010), which accelerates chlorophyll degradation, lowers leaf area index and depresses photosynthetic capacity (Lu et al., 2017). Meanwhile, heat stress elevates transpiration and respiration rates, suppresses photosynthetic and water-use efficiency, compromises cell membrane integrity and disturbs metabolic homeostasis, consequently limiting grain yield and quality formation (Lu et al., 2017; Urban et al., 2018; Posch et al., 2019). In addition, heat-induced metabolic perturbation promotes excessive intracellular reactive oxygen species (ROS) accumulation (Martineau et al., 1979; Wang et al., 2010; Zhou et al., 2014). Excess ROS disrupts biomembrane structure and function, exacerbates oxidative injury, increases membrane permeability and electrolyte leakage, and ultimately triggers irreversible cellular damage (Asada, 2006; Khan et al., 2019; Neves et al., 2019).

With progressive global climate change, heat events during the wheat growing season have become more frequent and intense. Heat stress mainly occurs during the later growth stage of wheat and is a key environmental factor that limits wheat yield and quality formation (Porter and Semenov, 2005; Arshad et al., 2018; Zhang et al., 2023). Numerous studies have demonstrated that temperatures exceeding 32 °C during grain filling substantially inhibit yield formation and deteriorate grain quality (Wollenweber et al., 2003; Yousuf et al., 2017). Therefore, exploring efficient strategies to enhance the late-stage heat tolerance of wheat is crucial for sustainable wheat production. In recent years, plant growth regulators (PGRs) are widely applied in agricultural production. Foliar PGR supplementation effectively alleviates heat-induced damage to wheat grain filling via optimizing plant architecture, enhancing stress tolerance and delaying leaf senescence, which ultimately improves wheat yield and quality. Consequently, PGR foliar application has emerged as a key agronomic strategy to mitigate high temperature stress and maintain stable wheat productivity under climate warming. Accordingly, this study investigated the regulatory effects of different PGRs on the heat tolerance and physiological characteristics of wheat at the late growth stage, and further explored the underlying physiological and molecular mechanisms of PGR-mediated heat tolerance. The objectives were to screen efficient heat-resistant PGRs, establish technical strategies for sustaining leaf function and improving wheat thermotolerance, and provide theoretical support and practical guidance for high-yield and high-quality wheat cultivation under heat stress.

## 2 Materials and methods

### 2.1 Plant material and growing conditions

Field experiments were carried out during the 2024 and 2025 winter wheat growing seasons at the Ruifeng Farmers’ Professional Cooperative in Huantai County, Zibo City, Shandong Province, China (36°59’20.88” N, 118°67’27.21” E). This study used the semi-winter wheat hybrid cultivar ‘Luyan 951’ as plant material. Jointly developed by the Crop Research Institute of Shandong Academy of Agricultural Sciences and Shandong Luyan Agricultural Seed Co., Ltd., this cultivar was derived from the cross of female parent ‘Luyuan 502’ and male parent ‘Jimai 22’. With a growth period of 236 days, ‘Luyan 951’ possesses superior agronomic traits, including high yield potential, strong cold tolerance, lodging resistance, broad-spectrum disease resistance, and drought tolerance, and is widely planted in the Huang-Huai-Hai Plain, China. The dynamic variations in field temperature from wheat anthesis to maturity across the two growing seasons are illustrated in **Figure 1**. Before sowing, the fertility of topsoil (0–20 cm) in the experimental field was determined. The soil bulk density was 1.56 g cm^-3^, with organic matter, total nitrogen, and alkali-hydrolyzable nitrogen contents of 16.86 g kg^-1^, 1.05 g kg^-1^, and 64.38 mg kg^-1^, respectively. The contents of total phosphorus, available phosphorus, total potassium, and available potassium were 6.37 g kg^-1^, 23.1 mg kg^-1^, 5.47 g kg^-1^, and 114.64 mg kg^-1^, respectively.

**Figure 1:**
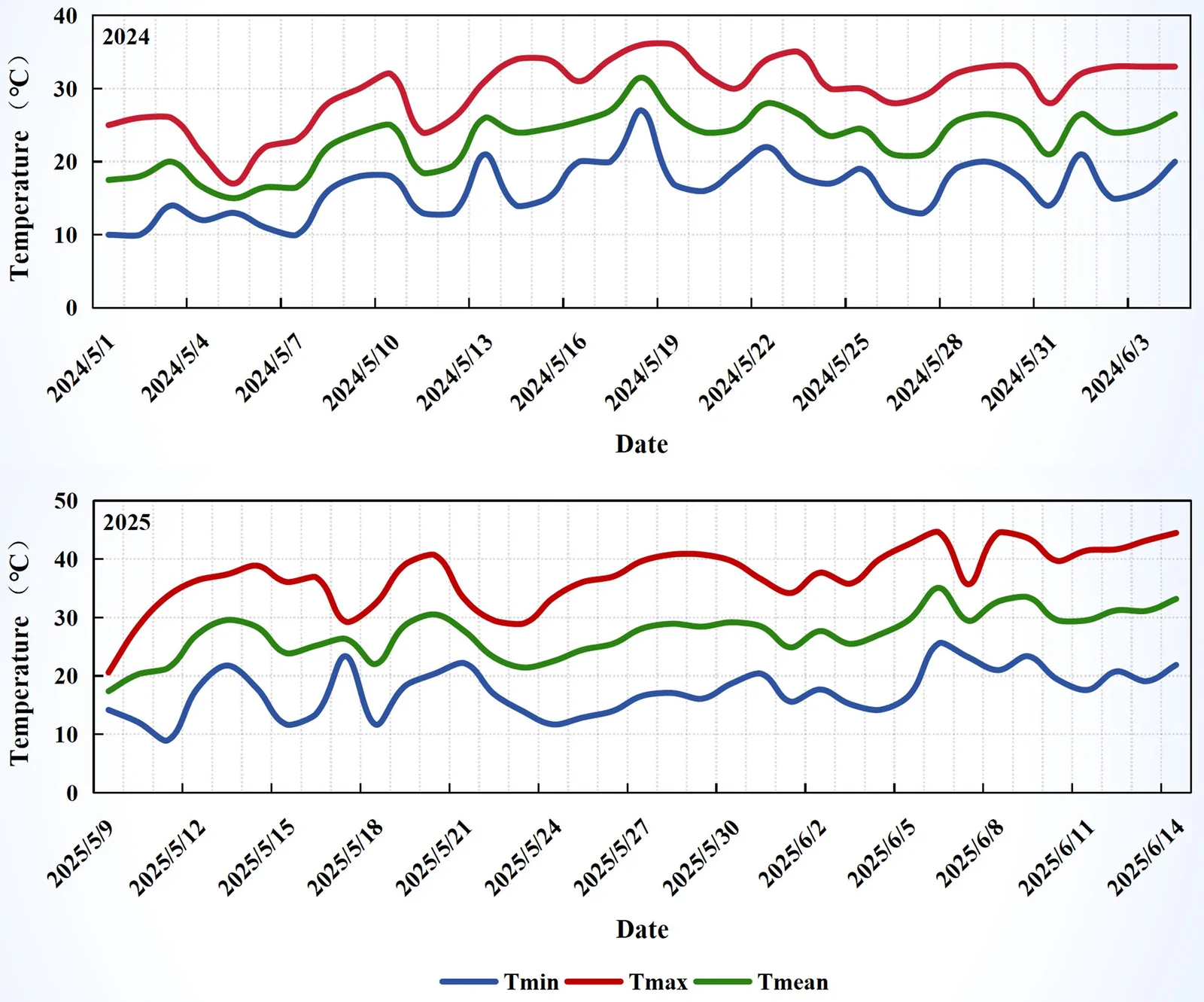
Dynamic records of temperature data of wheat after anthesis in 2024 and 2025.

### 2.2 Experimental design

Wheat seeds were sown in uniform rows with a row spacing of 18 cm, and the planting density was set at 8.25 million plants per hectare. Each experimental plot covered an area of 160 m² (16 m × 10 m). A total of five treatments were established in this field trial: (1) Control (CK), foliar application of distilled water; (2) EBR, foliar application of 0.1 µmol L^-1^ 2,4-epibrassinolide; (3) MT, foliar application of 100 µmol L^-1^ melatonin; (4) K, foliar application of 30 mmol L^-1^ KH_2_PO_4_; (5) Mix, foliar application of 0.05 µmol L^-1^ 2,4-epibrassinolide + 50 µmol L^-1^ melatonin + 15 mmol L^-1^ KH_2_PO_4_. Foliar spraying was performed twice at the booting and flowering stages, respectively. All solutions were uniformly sprayed on both adaxial and abaxial leaf surfaces using a knapsack sprayer, with a consistent application volume of 750 kg hm^-2^ for each spraying event to ensure full leaf wetness. The experiment was arranged in a randomized complete block design with three biological replicates for each treatment. All other field management practices were implemented in accordance with local conventional wheat cultivation protocols.

### 2.3 Measurement of wheat yield and its constituent factors

At wheat maturity, wheat ears within a 1 m^2^ area were manually harvested from each plot, and the spike number per unit area was recorded for each treatment. After threshing, a 200 g subsample of grains was collected from each plot to determine grain moisture content. The remaining grain samples were naturally air-dried to calculate the final grain yield per hectare. For yield component analysis, twenty representative spikes were randomly selected per treatment to quantify the grain number per spike. In addition, one thousand intact grains were randomly counted and weighed after oven drying using a seed counter to determine the 1000-grain weight. Experiments were performed using three independent biological replicates. Statistical analysis was subsequently conducted to evaluate the regulatory effects of different plant growth regulator applications on yield components of winter wheat.

### 2.4 Determination of dry matter content of different organs of wheat

At wheat maturity, twenty representative plants were randomly harvested from each treatment. The sampled plants were divided into three above-ground components: leaves, stems, and spikes. All plant tissues were first deactivated at 105 °C for 30 min, followed by oven-drying at 65 °C until a constant weight was achieved. The dry weight of each organ was individually measured to evaluate the effects of exogenous plant growth regulators on above-ground dry matter accumulation and its partitioning in wheat. All sampling and determination procedures were performed with three independent biological replicates per treatment.

### 2.5 Measurement of photosynthetic characteristics and leaf area of wheat

On the 20th day after anthesis, gas exchange parameters of wheat flag leaves were measured using a portable LI-6400 photosynthetic system (LI-COR, USA). All determinations were performed from 9:30 to 11:00 a.m. under stable natural sunlight conditions. For each experimental plot, uniformly grown, healthy, and disease-free wheat plants without mechanical damage were randomly selected. Four key photosynthetic indices, including net photosynthetic rate (Pn), stomatal conductance (Gs), intercellular CO_2_ concentration (Ci), and transpiration rate (Tr), were quantified following the method previously described by Zhang et al. (2022). Furthermore, the length and width of fully expanded flag leaves were measured with a straightedge for leaf area calculation, and the relative chlorophyll content (SPAD value) was determined using a handheld SPAD-502 Plus chlorophyll meter. Only intact and fully expanded flag leaves were adopted for all physiological measurements. Each treatment was set up with three independent biological replicates to ensure the reliability and accuracy of experimental data.

### 2.6 Determination of indicators of the antioxidant defense system of wheat

On the 20th day after anthesis, healthy and uniformly growing wheat plants were randomly selected from each experimental plot. Fresh flag leaf samples were harvested and immediately frozen in liquid nitrogen for subsequent quantification of antioxidant enzyme activities and non-enzymatic antioxidant substances associated with plant antioxidant defense systems. Fresh wheat flag leaf samples (0.50 g) were ground in a pre-cooled mortar with 5 mL phosphate buffer (pH 7.8) under ice-bath conditions. The homogenate was centrifuged, and the resulting supernatant was transferred to test tubes and stored at 0-4 °C. Commercial kits from Jiancheng Bioengineering Institute (Nanjing, Jiangsu, China) were used in this assay. According to the manufacturer’s standard operating procedures, the absorbance of wheat flag leaf extracts from different treatments was measured at 405 nm and 550 nm, respectively, to calculate hydrogen peroxide (H_2_O_2_) content and superoxide anion (O_2_^-^) generation rate.

The activities of superoxide dismutase (SOD), peroxidase (POD), and catalase (CAT) were assayed spectrophotometrically following the protocols reported by Wei et al. (2022). For the SOD assay, the reaction mixture containing nitroblue tetrazolium, methionine, riboflavin, and EDTA-Na_2_ was combined with 200 μL enzyme extract, and absorbance was determined at 560 nm. For the POD assay, 100 μL enzyme extract was mixed with 3 mL guaiacol reaction solution in a cuvette. Absorbance was recorded at 470 nm at 1 min intervals for three measurements, and POD activity was expressed as the absorbance change per minute. For the CAT assay, 3 mL of 20 mmol·L^-1^ H_2_O_2_ solution was mixed with 100 μL enzyme extract. Absorbance at 240 nm was measured 15 s after mixing, and data were collected every 30 s for six time points. Malondialdehyde (MDA) content was determined as described by Wei et al. (2022). Briefly, 1 mL enzyme extract was mixed with 1 mL phosphate buffer (pH 7.8), followed by the addition of 4 mL thiobarbituric acid solution. The mixture was boiled for 15 min, rapidly cooled, and centrifuged, and absorbance was measured at 450, 532, and 600 nm.

The contents of ascorbic acid (ASA) and reduced glutathione (GSH) were determined spectrophotometrically according to Li et al. (2020). For ASA assay, fresh wheat flag leaf samples (5.00 g) were homogenized with 20 mL of 50 g L^-1^ trichloroacetic acid on ice. The homogenate was brought to 100 mL with the same extractant and incubated for 10 min. An aliquot (1 mL) of the extract was sequentially mixed with 1 mL trichloroacetic acid, 1 mL absolute ethanol, 0.5 mL 0.4% phosphoric acid ethanol, 1 mL BP-ethanol, and 1 mL FeCl_3_-ethanol solutions, and absorbance was detected at 534 nm. For GSH assay, fresh wheat flag leaf samples (2.50 g) were homogenized in 5 mL of 50 g L^-1^ trichloroacetic acid on ice, followed by centrifugation to collect the supernatant. The blank, consisting of 1 mL distilled water, 1 mL 0.1 mol L^-1^ phosphate buffer (pH 7.7) and 0.5 mL 4 mmol L^-1^ DTNB solution, was used to zero the spectrophotometer at 412 nm. Two reaction tubes containing 1 mL supernatant and 1 mL phosphate buffer (pH 7.7) were prepared; 0.5 mL 4 mmol L^-1^ DTNB solution was added to one tube, and 0.5 mL phosphate buffer (pH 6.8) to the other. After incubation at 25 °C for 10 min, absorbance was measured at 412 nm. All physiological measurements were performed with three independent biological replicates for each treatment.

### 2.7 Determination of transcriptome data and bioinformatics analysis

At 20 days after anthesis, healthy wheat plants with uniformly growth were randomly selected from each plot, and the flag leaves were collected as test samples. All leaf samples were immediately frozen in liquid nitrogen and stored at -80 °C for subsequent transcriptome sequencing, with three independent biological replicates performed in each treatment. Total RNA was extracted from flag leaf samples using a TRIzol reagent kit (Invitrogen, Carlsbad, CA, USA) according to the manufacturer’s instructions. RNA quality was verified by RNase-free agarose gel electrophoresis, and RNA integrity was assessed using an Agilent 2100 Bioanalyzer (Agilent Technologies, Palo Alto, CA, USA). The mRNA was enriched using mRNA Capture Beads, purified, and fragmented under high temperature conditions. The fragmented mRNA was then used as a template to synthesize the first strand of cDNA in a reverse transcription enzyme mixture system. While synthesizing the second strand of cDNA, end repair and A-tailing were completed. Next, adapters were ligated, and Hieff NGS^®^ DNA Selection Beads were used for purification to select target fragments. PCR library amplification was then performed, and finally, detection was carried out using the Illumina NovaSeq X Plus (Gene Denovo Biotechnology Co., Guangzhou, China).

Raw sequencing reads were preprocessed and filtered to obtain high-quality clean reads by removing adapter-contaminated reads, reads with ambiguous N bases exceeding 10%, and low-quality reads with more than 50 bases at Q-value ≤ 20. Raw reads were aligned to the ribosomal RNA (rRNA) database using Bowtie2, and rRNA-mapped reads were subsequently removed. The remaining clean reads were adopted for transcript assembly and gene abundance calculation. A reference genome index was constructed, and paired-end clean reads were mapped to the reference genome via HISAT2 (v2.1.0) with default parameters. Transcript assembly for mapped reads of each sample was performed using StringTie (v1.3.1) based on the wheat reference genome. The expression abundance and variation of each transcript were quantified as fragments per kilobase of transcript per million mapped reads (FPKM) using RSEM software. RNAs differential expression analysis was performed by DESeq2 software between two different groups. Genes with a false discovery rate (FDR) ≤ 0.05 and absolute fold change ≥ 2 were identified as differentially expressed genes (DEGs). Functional enrichment and metabolic-pathway annotation of DEGs were conducted against the Kyoto Encyclopedia of Genes and Genomes (KEGG) database (http://www.genome.jp/kegg/). P-values derived from KEGG enrichment analysis were adjusted using the false-discovery-rate (FDR) correction method, and pathways with FDR ≤ 0.05 were regarded as significantly enriched.

### 2.8 Statistical analysis

Statistical data sorting was completed in Microsoft Excel 2021 (Microsoft Corp., Redmond, WA, USA). One-way analysis of variance (ANOVA) was implemented via IBM SPSS Statistics 22.0 (IBM Corp., Armonk, NY, USA), and pairwise comparisons were carried out using the Tukey’s HSD (Honestly Significant Difference) test at a threshold of p < 0.05. All figures were plotted with Origin 2023 (OriginLab Inc., Northampton, MA, USA). All data presented in figures are expressed as mean ± standard error (SE) from three independent biological replicates. The gene expression heatmap was generated with the online analytical platform OmicShare (https://www.omicshare.com).

## 3 Results

### 3.1 Effects of different plant growth regulators on wheat yield and yield components under natural high temperature stress

The phenotypic traits of wheat plants in response to foliar application of different plant growth regulators are presented in **Figure 2**. Data obtained from the 2024 and 2025 growing seasons indicated that foliar spraying of different plant growth regulators had no significant effect on wheat spike number (**Figure 3**). In 2024, spike grain number was significantly increased by 30.77%, 29.61%, 24.43%, and 32.43% under K, EBR, MT, and Mix treatments, respectively, relative to the control (CK). Moreover, EBR and MT treatments increased wheat 1000-grain weight by 2.87% and 4.07%, respectively. Grain yield was also markedly improved by 33.19% (EBR), 30.21% (MT), 33.02% (K), and 32.62% (Mix) in 2024. Consistent yield-promoting responses were observed in 2025. Compared with CK, the Mix treatment significantly elevated 1000-grain weight and grain yield by 3.31% and 9.42%, respectively. Two-year field data demonstrated that foliar regulator application exerted no significant effect on wheat spike number. Collectively, the two-year field results indicate that foliar application of appropriate plant growth regulators improves wheat grain yield mainly by increasing spike grain number and 1000-grain weight, the two decisive components of wheat yield.

**Figure 2:**
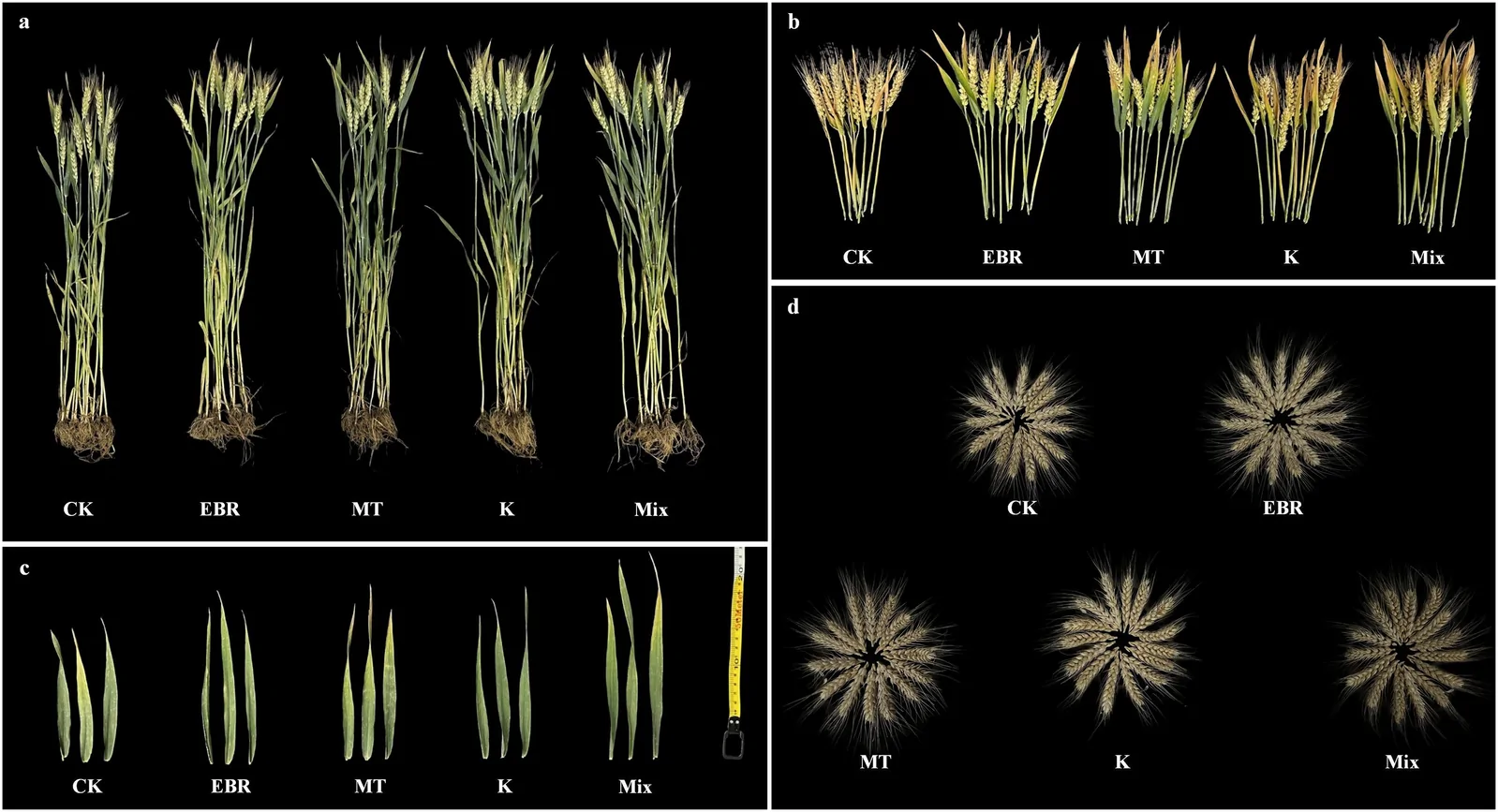
The regulatory effect of different plant growth regulators on wheat plant phenotype under natural high temperature stress. **(a)** Whole wheat plants; **(b)** Wheat spikes during grain filling; **(c)** Wheat flag leaves; **(d)** Wheat spikes at maturity. CK, distilled water; EBR, 0.1 µmol L^-1^ 2,4-epibrassinolide; MT, 100 µmol L^-1^ melatonin; K, 30 mmol L^-1^ KH_2_PO_4_; Mix, 0.05 µmol L^-1^ 2,4-epibrassinolide + 50 µmol L^-1^ melatonin + 15 mmol L^-1^ KH_2_PO_4_.

**Figure 3:**
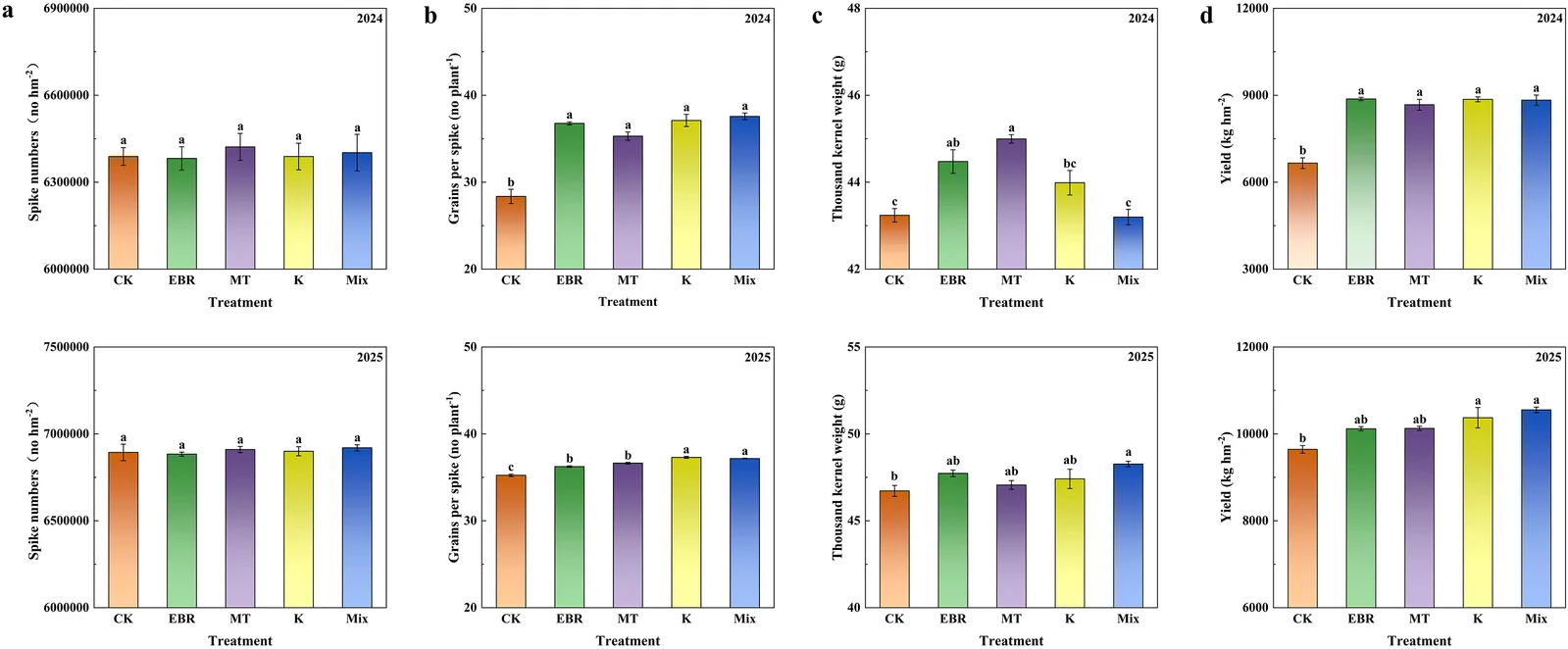
Effects of different plant growth regulators on wheat yield and yield components under natural high temperature stress. **(a)** Spike numbers; **(b)** Grain number per spike; **(c)** Thousand kernel weight; **(d)** Grain yield. CK, distilled water; EBR, 0.1 µmol L^-1^ 2,4-epibrassinolide; MT, 100 µmol L^-1^ melatonin; K, 30 mmol L^-1^ KH_2_PO_4_; Mix, 0.05 µmol L^-1^ 2,4-epibrassinolide + 50 µmol L^-1^ melatonin + 15 mmol L^-1^ KH_2_PO_4_. Data represent mean ± standard error (SE) from triplicate biological replicates. To compare the means, the Tukey’s HSD (Honestly Significant Difference) test was computed using analysis of variance (ANOVA). Different letters represent significant differences at p < 0.05.

### 3.2 Effects of different plant growth regulators on dry matter accumulation and distribution of wheat under natural high temperature stress

As shown in **Figure 4**, foliar application of plant growth regulators significantly promoted above-ground dry matter accumulation in wheat during the 2024 growing season. Relative to the CK treatment, EBR, MT, K, and Mix treatments significantly increased single-plant leaf dry matter by 46.83%, 52.38%, 49.21%, and 48.41%, and elevated spike dry matter by 10.31%, 12.35%, 7.96%, and 8.98%, respectively. All regulator treatments substantially improved total aboveground dry matter accumulation, which increased by 11.61% (EBR), 12.82% (MT), 13.41% (K), and 13.95% (Mix) relative to CK. Consistent promotional trends were observed in the 2025 growing season. Relative to CK, the EBR, MT, K, and Mix treatments significantly increased single-plant leaf dry matter by 17.03%, 32.88%, 40.81%, and 60.99% and main stem dry matter by 13.16%, 6.74%, 11.37%, and 11.93%, respectively. Meanwhile, all treatments markedly elevated total aboveground dry matter by 6.89%, 5.60%, 9.81%, and 11.43%, correspondingly.

**Figure 4:**
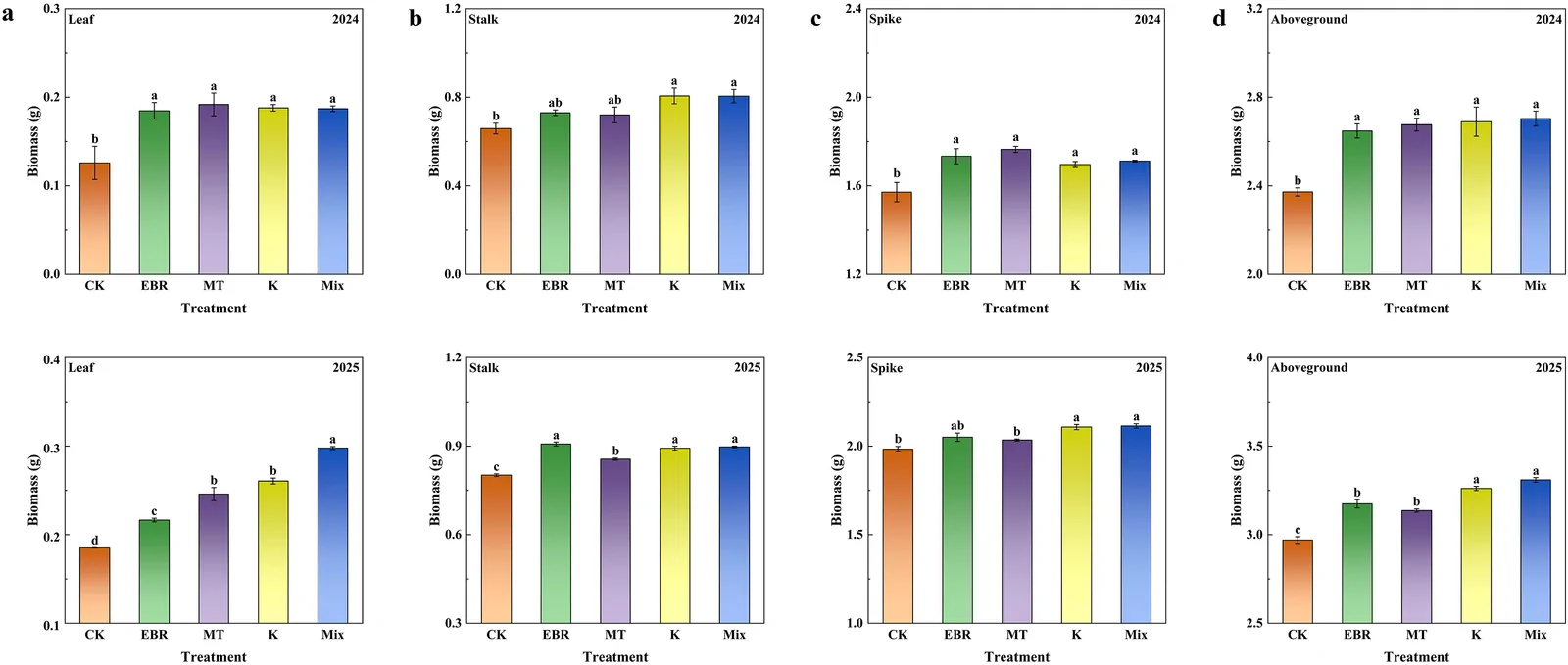
Effects of different plant growth regulators on dry matter accumulation and distribution of wheat under natural high temperature stress. **(a)** Leaf dry weight; **(b)** Stem dry weight; **(c)** Spike dry weight; **(d)** Aboveground dry weight. CK, distilled water; EBR, 0.1 µmol L^-1^ 2,4-epibrassinolide; MT, 100 µmol L^-1^ melatonin; K, 30 mmol L^-1^ KH_2_PO_4_; Mix, 0.05 µmol L^-1^ 2,4-epibrassinolide + 50 µmol L^-1^ melatonin + 15 mmol L^-1^ KH_2_PO_4_. Data represent mean ± standard error (SE) from triplicate biological replicates. To compare the means, the Tukey’s HSD (Honestly Significant Difference) test was computed using analysis of variance (ANOVA). Different letters represent significant differences at p < 0.05.

### 3.3 Effects of different plant growth regulators on flag leaf photosynthetic characteristics and leaf area of wheat under natural high temperature stress

To elucidate the regulatory effects of different plant growth regulators on wheat tolerance to natural high-temperature stress after anthesis, we determined leaf relative chlorophyll content (SPAD value), photosynthetic gas exchange parameters, flag leaf area, and single-plant total leaf area under all treatments. As shown in **Figure 5**, foliar application of plant growth regulators significantly improved wheat leaf SPAD values across experimental treatments. In 2024, EBR, MT, K, and Mix treatments significantly increased flag leaf area by 25.71%, 51.14%, 42.26%, and 30.25%, and elevated single-plant total leaf area by 23.39%, 45.32%, 44.52%, and 41.20%, respectively, compared with CK. In 2025, flag leaf area showed no significant differences between CK and the EBR, MT, and K treatments, while the Mix treatment markedly increased flag leaf area by 26.75%. Furthermore, all regulator treatments significantly increased single-plant total leaf area in 2025, with no significant differences observed among the four treatments.

**Figure 5:**
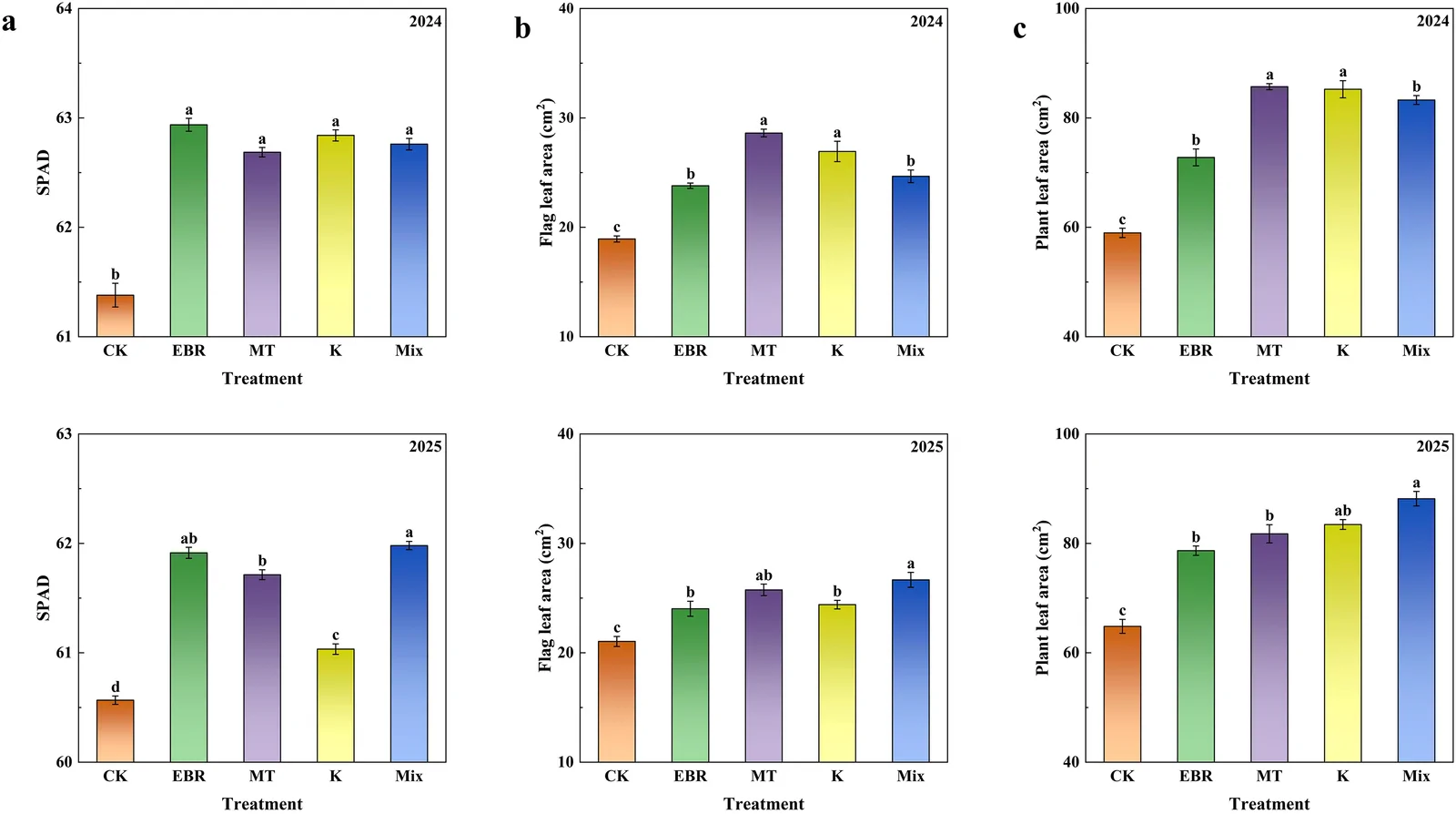
Effects of different plant growth regulators on SPAD value and leaf area of wheat flag leaves under natural high temperature stress. **(a)** SPAD value; **(b)** Flag leaf area; **(c)** Whole-plant leaf area. CK, distilled water; EBR, 0.1 µmol L^-1^ 2,4-epibrassinolide; MT, 100 µmol L^-1^ melatonin; K, 30 mmol L^-1^ KH_2_PO_4_; Mix, 0.05 µmol L^-1^ 2,4-epibrassinolide + 50 µmol L^-1^ melatonin + 15 mmol L^-1^ KH_2_PO_4_. Data represent mean ± standard error (SE) from triplicate biological replicates. To compare the means, the Tukey’s HSD (Honestly Significant Difference) test was computed using analysis of variance (ANOVA). Different letters represent significant differences at p < 0.05.

Gas exchange parameters of flag leaves further confirmed the regulatory effects of plant growth regulators on wheat photosynthetic capacity under high temperature stress after anthesis (**Table 1**). In 2024, compared with CK, the EBR, MT, and Mix treatments significantly increased the net photosynthetic rate (Pn) of flag leaves by 11.01%, 5.08%, and 12.44%, whereas the K treatment exerted no significant effect on Pn. Relative to CK, EBR and Mix treatments substantially enhanced flag leaf stomatal conductance (Gs) by 34.25% and 32.53%, increased intercellular CO_2_ concentration (Ci) by 5.24% and 6.67%, and elevated transpiration rate (Tr) by 8.77% and 11.71%, respectively. Similar positive effects on photosynthetic performance were observed in 2025. Notably, no significant differences in flag leaf Tr were detected between all regulator treatments and CK in 2025. Overall, exogenous plant growth regulators effectively improved wheat photosynthetic performance by increasing chlorophyll content, expanding leaf area, and enhancing leaf photosynthetic efficiency. These changes prolonged leaf green retention and ultimately improved wheat tolerance to high temperature stress after anthesis.

**Table 1:** Effects of different plant growth regulators on photosynthetic gas exchange parameters of wheat flag leaves under natural high temperature stress.

Treatment	Net photosynthesis	Stomatal conductance	Intercellular CO_2_ concentration	Transpiration rate
	(μmol CO_2_ m^-2^ s^-1^)	(mmol H_2_O m^-2^ s^-1^)	(µmol CO_2_ mol^-1^)	(mmol H_2_O m^-2^ s^-1^)
2024				
CK	18.18 ± 0.167c	0.53 ± 0.0009d	294.92 ± 1.216d	10.68 ± 0.036d
EBR	20.18 ± 0.197a	0.71 ± 0.0012a	310.38 ± 0.322b	11.62 ± 0.013b
MT	19.28 ± 0.027b	0.54 ± 0.0006c	291.81 ± 0.052e	10.78 ± 0.014d
K	18.57 ± 0.125c	0.54 ± 0.0009c	298.84 ± 0.534c	10.94 ± 0.033c
Mix	20.44 ± 0.109a	0.70 ± 0.0012b	314.61 ± 0.396a	11.93 ± 0.004a
2025				
CK	13.45 ± 0.136d	0.61 ± 0.0029c	344.65 ± 0.425d	12.31 ± 0.008ab
EBR	15.93 ± 0.074a	0.73 ± 0.0007a	357.92 ± 0.577b	12.29 ± 0.005ab
MT	15.15 ± 0.080b	0.73 ± 0.0003a	357.97 ± 0.396b	12.35 ± 0.052b
K	14.56 ± 0.120c	0.67 ± 0.0018b	352.79 ± 0.358c	12.16 ± 0.013ab
Mix	16.38 ± 0.075a	0.74 ± 0.0105a	362.31 ± 0.507a	12.40 ± 0.085a

CK, distilled water; EBR, 0.1 µmol L^-1^ 2,4-epibrassinolide; MT, 100 µmol L^-1^ melatonin; K, 30 mmol L^-1^ KH_2_PO_4_; Mix, 0.05 µmol L^-1^ 2,4-epibrassinolide + 50 µmol L^-1^ melatonin + 15 mmol L^-1^ KH_2_PO_4_. Data represent mean ± standard error (SE) from triplicate biological replicates. To compare the means, the Tukey’s HSD (Honestly Significant Difference) test was computed using analysis of variance (ANOVA). Different letters represent significant differences at p < 0.05.

### 3.4 Effects of different plant growth regulators on the contents of key antioxidant metabolites in wheat flag leaves under natural high temperature stress

To explore the effects of exogenous regulators on wheat antioxidant metabolism under high temperature stress after anthesis, key antioxidant metabolites in flag leaves were measured across all treatments (**Figure 6**). In 2024, the MT, K, and Mix treatments markedly decreased superoxide anion (O_2_^-^) production rate by 12.65%, 9.99%, and 24.49%, respectively. For non-enzymatic antioxidants, flag leaf glutathione (GSH) content was significantly elevated by MT (9.85%), K (13.97%), and Mix (13.43%) treatments. Additionally, EBR and K exerted no significant effects on ascorbic acid (AsA) content, whereas MT and Mix increased AsA levels by 13.66% and 20.49%, respectively. Similar antioxidant regulatory responses were observed in 2025 with partial interannual variation. Compared with CK, MT, K, and Mix decreased MDA content by 9.73%, 7.58%, and 11.25%, respectively. Similarly, H_2_O_2_ content remained unchanged under EBR, while declining by 16.62% (MT), 20.96% (K), and 20.21% (Mix). Moreover, the Mix treatment significantly increased GSH and AsA content by 21.25% and 30.11%, respectively. In summary, exogenous plant growth regulators alleviate oxidative damage caused by heat exposure in the flag leaves of wheat by reducing the accumulation of reactive oxygen species (ROS) and improving non-enzymatic antioxidant metabolism.

**Figure 6:**
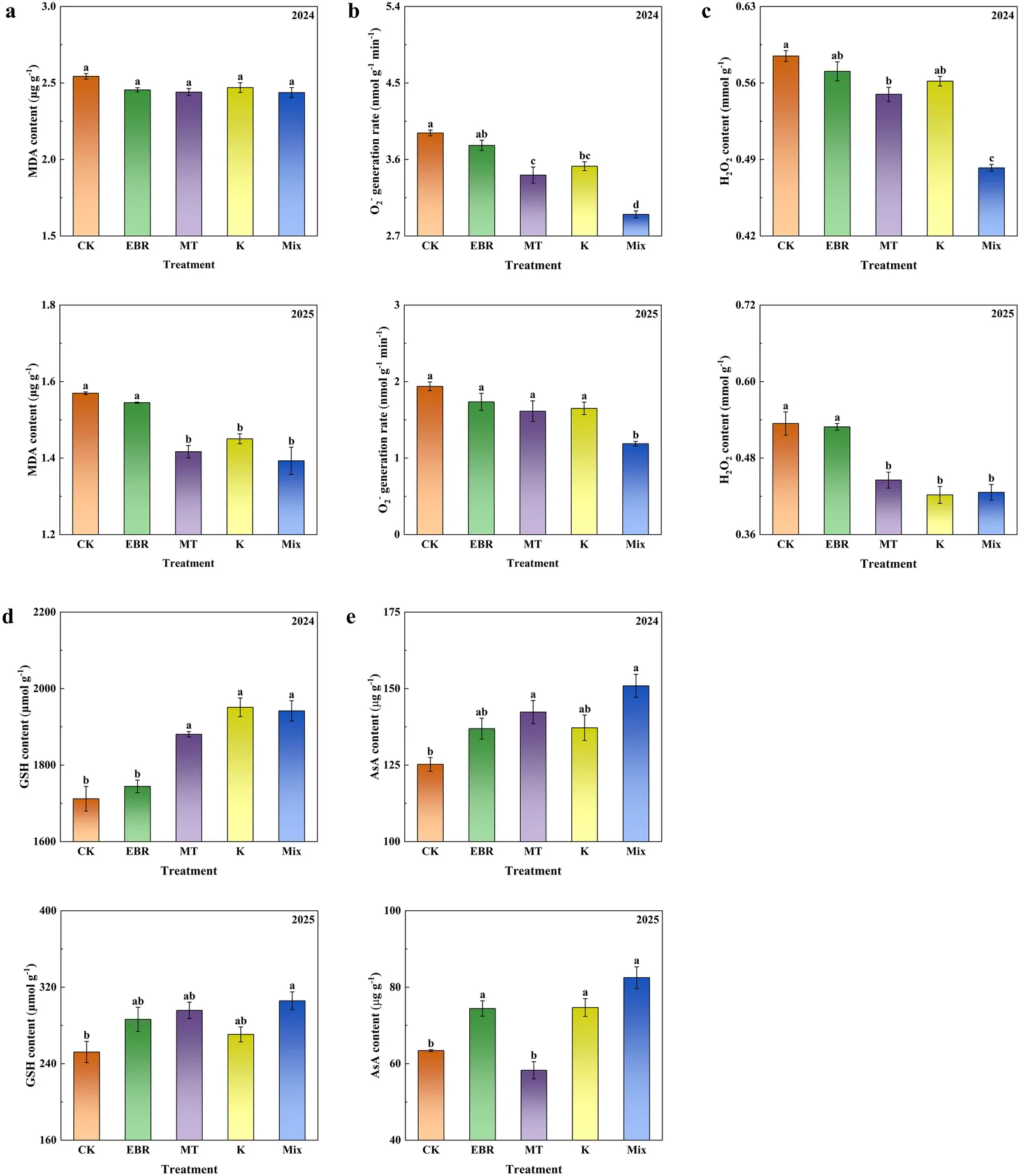
Effects of different plant growth regulators on the contents of key antioxidant metabolites in wheat flag leaves under natural high temperature stress. **(a)** MDA content; **(b)** O_2_^-^ generation rate; **(c)** H_2_O_2_ content; **(d)** GSH content; **(e)** AsA content. CK, distilled water; EBR, 0.1 µmol L^-1^ 2,4-epibrassinolide; MT, 100 µmol L^-1^ melatonin; K, 30 mmol L^-1^ KH_2_PO_4_; Mix, 0.05 µmol L^-1^ 2,4-epibrassinolide + 50 µmol L^-1^ melatonin + 15 mmol L^-1^ KH_2_PO_4_. Data represent mean ± standard error (SE) from triplicate biological replicates. To compare the means, the Tukey’s HSD (Honestly Significant Difference) test was computed using analysis of variance (ANOVA). Different letters represent significant differences at p < 0.05.

### 3.5 Effects of different plant growth regulators on the activities of key antioxidant metabolic enzymes in wheat flag leaves under natural high temperature stress

Antioxidant enzyme activities in wheat flag leaves were further quantified to clarify the enzymatic antioxidant defense mechanism regulated by exogenous plant growth regulators (**Figure 7**). In 2024, EBR, MT, K, and Mix treatments significantly increased superoxide dismutase (SOD) activity by 30.80%, 22.11%, 19.07%, and 47.72%, respectively, relative to CK. The activities of catalase (CAT), peroxidase (POD), glutathione peroxidase (GPX), and ascorbic acid peroxidase (APX) were also markedly elevated by the four treatments, with the Mix treatment exhibiting the maximum increase in all detected antioxidant enzyme activities. Consistent upregulation patterns of antioxidant enzymes were observed in 2025. All regulator treatments significantly increased flag leaf SOD and CAT activities compared with CK. POD activity showed no significant difference between EBR and CK, while MT, K, and Mix significantly increased POD activity by 28.59%, 35.45%, and 20.84%, respectively. For GPX activity, no statistical difference was detected between EBR, MT and CK, whereas K, and Mix upregulated GPX activity by 27.22%, and 31.77%. Additionally, EBR and MT exerted no significant effects on APX activity, while K and Mix significantly enhanced APX activity by 27.32% and 27.76%, respectively.

**Figure 7:**
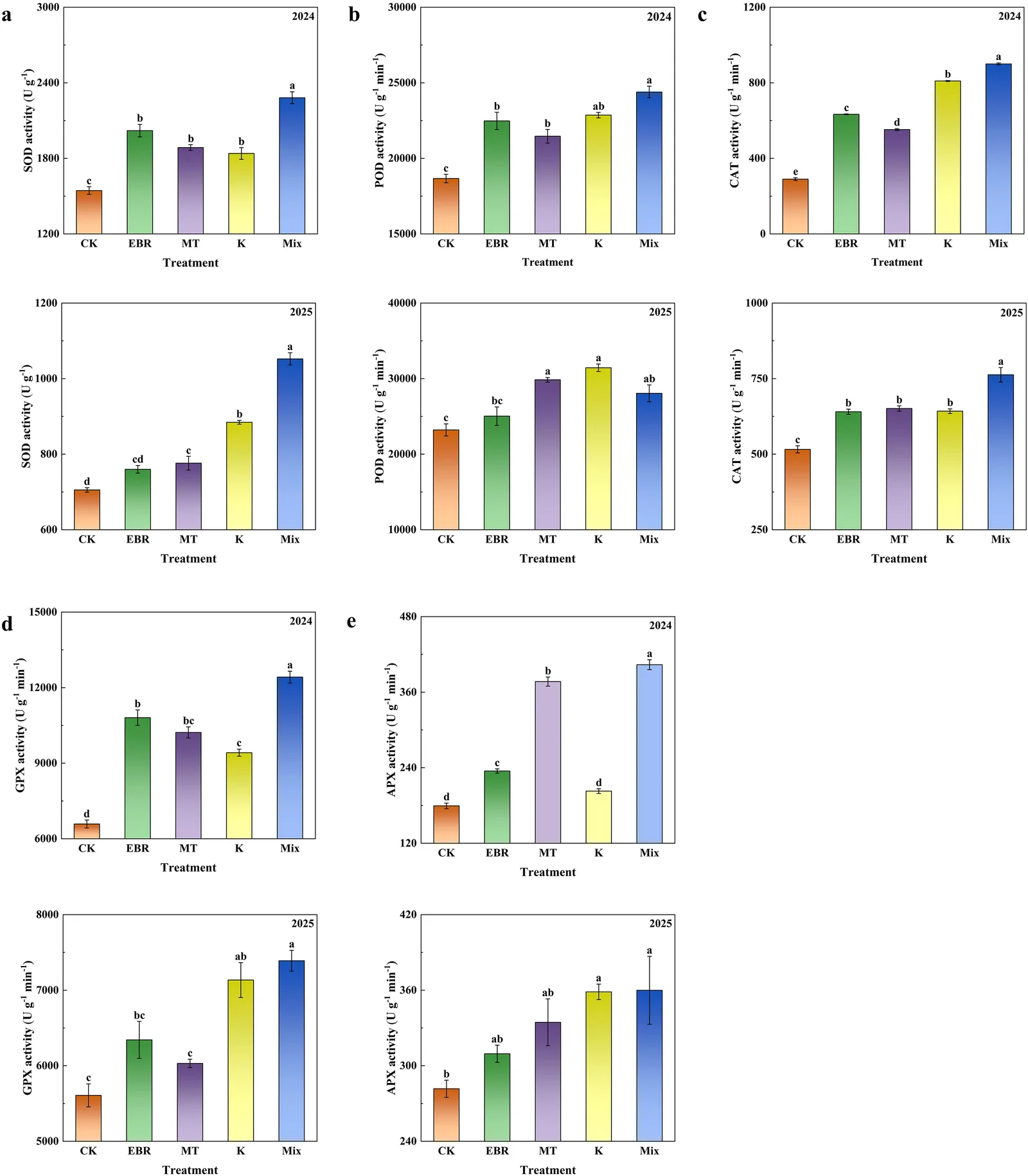
Effects of different plant growth regulators on the activities of key antioxidant metabolic enzymes in wheat flag leaves under natural high temperature stress. **(a)** SOD activity; **(b)** POD activity; **(c)** CAT activity; **(d)** GPX activity; **(e)** APX activity. CK, distilled water; EBR, 0.1 µmol L^-1^ 2,4-epibrassinolide; MT, 100 µmol L^-1^ melatonin; K, 30 mmol L^-1^ KH_2_PO_4_; Mix, 0.05 µmol L^-1^ 2,4-epibrassinolide + 50 µmol L^-1^ melatonin + 15 mmol L^-1^ KH_2_PO_4_. Data represent mean ± standard error (SE) from triplicate biological replicates. To compare the means, the Tukey’s HSD (Honestly Significant Difference) test was computed using analysis of variance (ANOVA). Different letters represent significant differences at p < 0.05.

### 3.6 Effects of different plant growth regulators on gene expression and key biological pathways in wheat flag leaves under natural high temperature stress

Given the prominent regulatory effects of plant growth regulators in alleviating natural high temperature stress after anthesis, including improved grain yield and optimized flag leaf physiological characteristics, transcriptome sequencing was performed on wheat flag leaves under EBR and MT treatments to further elucidate the transcriptional mechanisms underlying exogenous regulator-mediated regulation of core metabolic pathways. Flag leaf samples under EBR and MT treatments were collected at 5, 10, 15, and 20 days after anthesis for transcriptomic analysis. It is worth noting that the 20th day after anthesis coincided with the high temperature in the test area, and the largest number of differentially expressed genes (DEGs) were detected at this stage. Therefore, flag leaf transcriptomic data obtained at 20th day after anthesis were selected for further analysis. DEGs were screened based on the criteria of false discovery rate (FDR) < 0.05 and |log_2_(fold change, FC)| > 1. The volcano plots illustrated the number of DEGs in each comparison group (**Figure 8**). A total of 8000 DEGs (3156 upregulated and 4844 downregulated) were identified in the CK-vs-EBR group (**Figure 8a**), while 23665 DEGs (4310 upregulated and 19355 downregulated) were detected in the CK-vs-MT group (**Figure 8b**). Heat maps further visualized the expression patterns of these DEGs in EBR (**Figure 8c**) and MT (**Figure 8d**) treatments relative to CK. Kyoto Encyclopedia of Genes and Genomes (KEGG) enrichment analysis revealed that photosynthesis (ko00195), Calvin cycle (ko00710), and carbon metabolism (ko01200) were significantly enriched in both CK-vs-EBR and CK-vs-MT comparison groups (**Figures 8e, f**), indicating the critical roles of these three pathways in wheat flag leaf responses to natural high temperature stress after anthesis. Previous studies have confirmed that the ROS scavenging system serves as a core regulator of crop thermotolerance. Accordingly, we further explored ROS-scavenging-related metabolic pathways and found that numerous DEGs from both comparison groups were significantly enriched in the glutathione metabolism pathway. Based on these findings, this study conducted a systemic analysis of the transcriptional expression profiles of the genes involved in these four key pathways.

**Figure 8:**
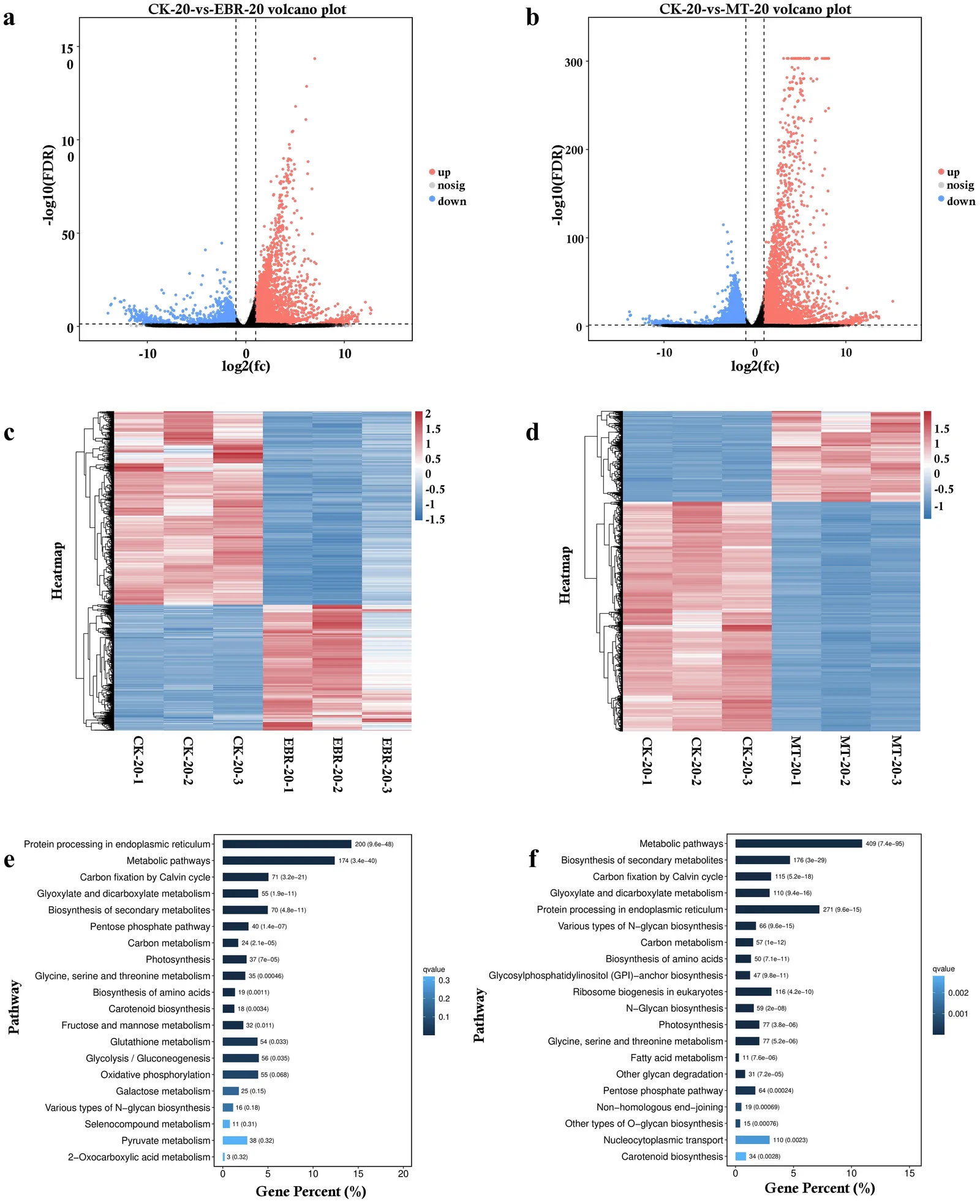
Differentially expressed genes and their key enriched biological pathways in flag leaves of wheat at 20 days after anthesis under EBR and MT treatments. **(a)** Volcano plot of DEGs between CK-vs-EBR; **(b)** Volcano plot of DEGs between CK-vs-MT; **(c)** Heatmap of expression levels of DEGs between CK-vs-EBR; **(d)** Heatmap of expression levels of DEGs between CK-vs-MT; **(e)** Key biological metabolic pathways enriched by DEGs between CK-vs-EBR; **(f)** Key biological metabolic pathways enriched by DEGs between CK-vs-MT. CK, distilled water; EBR, 0.1 µmol L^-1^ 2,4-epibrassinolide; MT, 100 µmol L^-1^ melatonin. The volcano plot shows DEGs numbers: red dots for upregulated genes, blue dots for downregulated genes, and black dots for genes with non-significant expression differences. The heatmap visualizes DEGs expression patterns, with columns representing samples and rows representing genes. Color gradients indicate gene expression levels, where red denotes high expression and blue denotes low expression.

In the CK-20 vs. EBR-20 comparison group, a total of 37 DEGs were annotated to the photosynthesis pathway (ko00195), encoding ATPF, PET, and PSB proteins. Meanwhile, 71 DEGs were enriched in the Calvin cycle pathway (ko00710), corresponding to MDH, MAE, GADPH, PRK, PGK, SBPase, PPC, FBP, GAPA, ALDO, and RBCS. In addition, 24 DEGs associated with carbon metabolism (ko01200) were identified, which encoded MDH, PFK, PYK, ALDO, CS, and GPI. A total of 54 DEGs were enriched in the glutathione metabolism pathway (ko00480), including genes encoding PGD, GPX, APX, GST, RRM, GGT, and GGCT. For the CK-20 vs. MT-20 group, 77 photosynthesis-related DEGs were annotated to the ATPF, PET, PSB, and PSA genes. The Calvin cycle pathway exhibited greater DEG enrichment, with 115 identified DEGs covering functional genes encoding MDH, GADPH, PRK, PGK, SBPase, PPC, RBC, ALDO, RPE, TPI, FBP, GAPA, GCAT, and GOT. Furthermore, 57 DEGs participated in carbon metabolism, corresponding to MDH, GAPDH, GLUD, PFK, PYK, ALDO, CS, GPI, and HPR. A total of 84 DEGs were enriched in the glutathione metabolism pathway, encoding PGD, GSR, GPX, APX, GST, RRM, GGT, and GGCT. Heat map visualization further revealed distinct expression levels of DEGs involved in the four key pathways under different treatments (**Figure 9**). Overall, foliar application of EBR and MT modulated the transcription of functional genes associated with photosynthesis, carbon metabolism, and glutathione metabolism in wheat flag leaves. Such transcriptional reprogramming of core metabolic pathways contributes to improved photosynthetic capacity and antioxidant defense efficiency, thereby alleviating heat-induced physiological damage in winter wheat under natural high temperature stress after anthesis.

**Figure 9:**
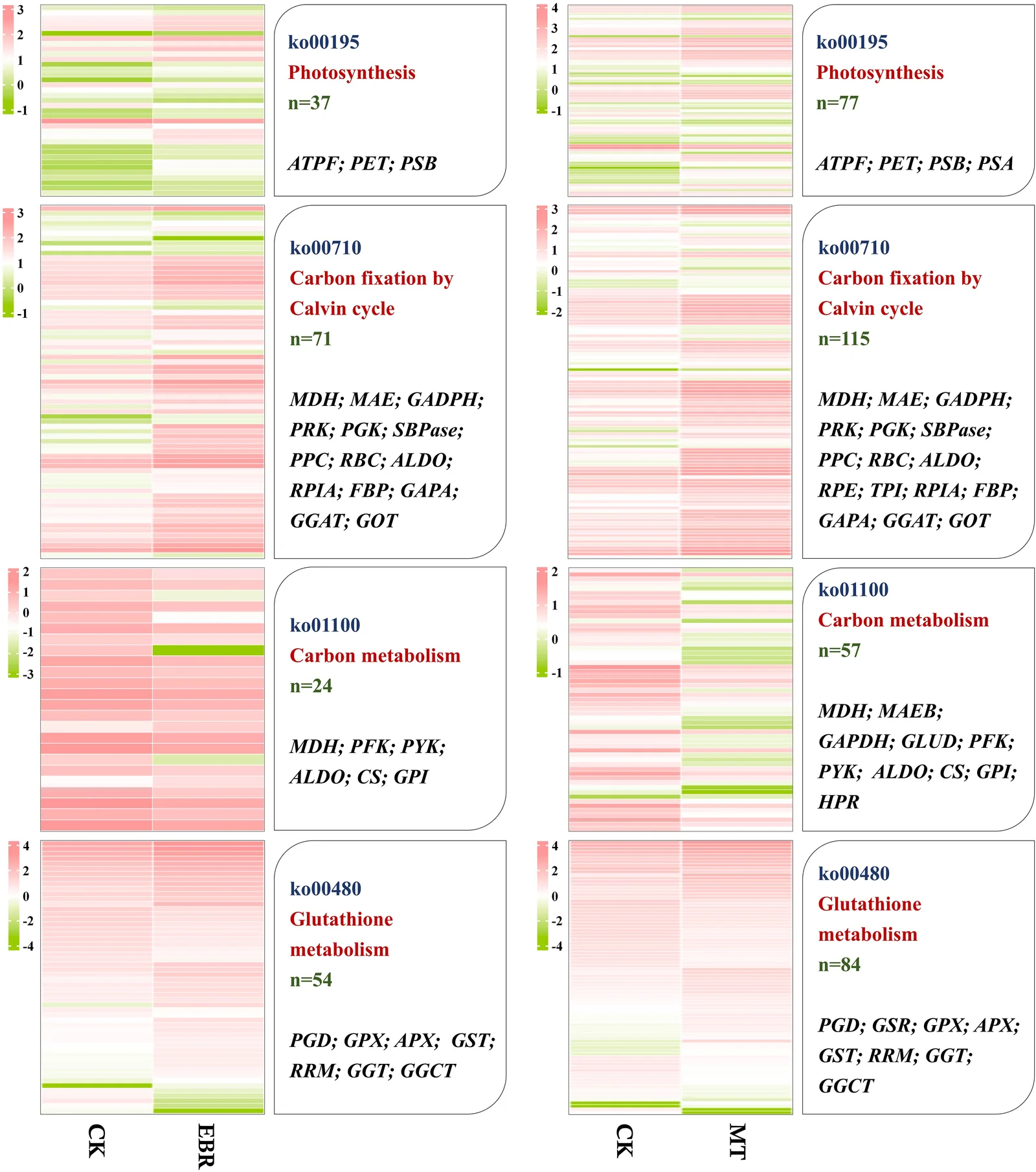
Key KEGG pathway and gene expression details of flag leaves in wheat at 20 days after anthesis under exogenous EBR and MT treatments. CK, distilled water; EBR, 0.1 µmol L^-1^ 2,4-epibrassinolide; MT, 100 µmol L^-1^ melatonin. ALDO, fructose-bisphosphate aldolase; APX, L-ascorbate peroxidase; ATPF, ATP synthase subunit; CS, citrate synthase; FBP, fructose-1,6-bisphosphatase; GADPH, glyceraldehyde-3-phosphate dehydrogenase; GAPA, glyceraldehyde-3-phosphate dehydrogenase A; GGAT, glutamate--glyoxylate aminotransferase; GGCT, γ-glutamyl cyclotransferase; GGT, γ-glutamyl transferase; GLUD, glutamate dehydrogenase; GOT, aspartate aminotransferase; GPI, hypothetical protein; GPX, glutathione peroxidase; GSR, glutathione reductase; GST, glutathione S-transferase; HPR, hydroxypyruvate reductase; MAE, NADP-dependent malic enzyme; MDH, malate dehydrogenase; PET, ferredoxin; PFK, phosphofructokinase; PGD, 6-phosphogluconate dehydrogenase; PGK, phosphoglycerate kinase; PPC, phosphoenolpyruvate carboxylase; PRK, phosphoribulokinase; PSA, photosystem I reaction center subunit; PSB, photosystem II reaction center PSB protein; PYK, Pyruvate kinase; RBC, ribulose bisphosphate carboxylase; RPE, ribulose-phosphate 3-epimerase; RRM, Ribonucleotide reductase subunit; SBPase, sedoheptulose-1,7-bisphosphatase; TPI, triosephosphate isomerase. The color in the heatmap indicates the gene expression. Red represents high expression, and blue represents low expression.

## 4 Discussion

### 4.1 Regulatory effects of exogenous application of different plant growth regulators on yield and dry matter accumulation of wheat under natural high temperature stress

Global climate warming has progressively increased the frequency and intensity of high temperature stress worldwide, posing a severe global threat to wheat growth, metabolic processes, grain formation, and final yield and quality (Lobell et al., 2011; White et al., 2012; Duan et al., 2025). Under such changing agroclimatic conditions, enhancing crop systemic resilience, mitigating environmental stress-induced damage, and safeguarding food security have become core challenges for global agricultural sustainability (Godfray et al., 2010; IPCC, 2019; Collins and Chenu, 2021; Mahaut et al., 2022). In the winter wheat production areas of China’s Huang-Huai-Hai Plain, the susceptibility of winter wheat to short-term extreme high temperature events has increased continuously. High temperature stress during the grain-filling stage acts as the predominant abiotic constraint on wheat productivity, as it curtails the grain-filling process and triggers substantial yield losses (Barnabás et al., 2010; Zheng et al., 2012, 2016; Ju et al., 2021). In this context, comprehensive evaluation of temperature regulatory effects on winter wheat growth and development is crucial for predicting crop performance under future climate scenarios.

Previous studies have quantified the temperature response of wheat grain filling, demonstrating that within the optimal temperature range of 15.8-27.7 °C, each 1 °C rise in ambient temperature shortens the grain-filling duration by three days and reduces single grain weight by 2.8 mg (Chowdhury and Wardlaw, 1978). Complementary evidence further confirmed stage-specific heat damage: heat stress at the early grain-filling stage primarily limits final yield by reducing grain number per spike, whereas stress occurring during the mid and late filling stages predominantly impairs grain weight formation. Such differential responses can be explained by heat-induced leaf and grain overheating, leaf wilting, and reduced grain water content, which collectively constrain grain biomass accumulation (Jenner, 1994; Viswanathan et al., 2001; Mirosavljević et al., 2021). Foliar fertilization has been reported to improve leaf photosynthetic capacity during the late grain-filling stage, increase spike grain number and 1000-grain weight, raise final grain yield, and optimize grain quality (Luo et al., 2014). Consistent with these previous results, our two-year field experiment showed that foliar application of different plant growth regulators exerted no significant effect on spike number of winter wheat under natural high temperature stress after anthesis. Nevertheless, all regulator treatments substantially increased spike grain number and 1000-grain weight. These findings indicate that the improvements in spike grain number and 1000-grain weight represent the primary basis for yield enhancement induced by exogenous plant growth regulators in winter wheat. Such yield-promoting effects of exogenous growth regulators have been extensively documented across various crops, including rice (Islam et al., 2019; Yao et al., 2020), maize (Niu et al., 2021), and wheat (Lv et al., 2017; Li et al., 2023a; Verma et al., 2024). Dry matter accumulation, distribution and translocation form the material basis for wheat yield formation. Grain-filling heat stress accelerates effective temperature accumulation, shortens the grain-filling period, and inhibits dry matter assimilation and translocation to grains, thus limiting wheat yield potential (Sadras and Monzon, 2006; Bala et al., 2021; Durbha et al., 2024). Exogenous potassium dihydrogen phosphate improves crop thermotolerance and promotes grain weight accumulation under abiotic stress (Yang et al., 2022). In this study, foliar application of EBR, MT, potassium fertilizer, and their mixture significantly increased leaf, main stem and spike dry matter mass, elevating total above-ground dry matter by 6.89%, 5.60%, 9.81%, and 11.43%, respectively, compared with the control. The superior performance of wheat grain yield and dry matter accumulation under the Mix treatment may be attributed to the integrated effects jointly regulated by multiple exogenously applied growth regulators. These findings confirm that foliar PGR application enhances above-ground dry matter accumulation in heat-stressed wheat. There is a similar relationship between the accumulation of dry matter after maize flowering and grain yield (Niu et al., 2021; Liu et al., 2022; Li et al., 2024), further supporting that PGRs mitigate heat injury by maintaining photosynthate accumulation and dry matter partitioning as a core physiological mechanism underlying yield preservation under natural high temperature stress after anthesis.

### 4.2 Regulatory effects of exogenous plant growth regulator application on photosynthetic physiological response mechanisms of wheat under natural high temperature stress

Wheat grain yield is jointly determined by “the source-sink” relationship, wherein photosynthetic source capacity, sink storage potential, and photosynthate translocation efficiency collectively govern yield formation. As the main photosynthetic organ of wheat, the flag leaf acts as the predominant source of grain assimilates, supplying up to 80% of grain carbohydrates during the mid-to-late grain-filling stages (Christopher et al., 2008; Li et al., 2021). Nevertheless, high temperature stress after anthesis markedly impairs source function by accelerating flag leaf senescence, promoting chloroplast degradation, and decreasing chlorophyll content, stomatal conductance, and transpiration rate (Mittler et al., 2012; Ru et al., 2023). These physiological disturbances reduce leaf photosynthetic efficiency and assimilate accumulation, hinder light energy absorption and conversion, and eventually result in insufficient grain filling and yield loss (Parry et al., 2011; Prasad and Djanaguiraman, 2014). Accordingly, delaying heat-triggered leaf senescence, extending the functional green period of photosynthetic leaves, and preserving flag leaf physiological activity during grain filling represent key strategies to stabilize wheat yield under high temperature stress. Chloroplast dysfunction and depressed photosystem efficiency constitute the primary constraints on photosynthetic rate under heat stress (Salvucci and Crafts-Brandner, 2004; Wang et al., 2012). In this study, foliar application of exogenous plant growth regulators significantly increased leaf SPAD values and expanded both flag leaf area and total plant leaf area. Meanwhile, EBR, MT, and Mix treatments substantially elevated the net photosynthetic rate of heat-stressed flag leaves. Prior research has confirmed that high temperature suppresses photosynthesis by reducing chlorophyll accumulation and depressing PSII activity, disrupting the electron transport chain and limiting carbon fixation (Mathur et al., 2014). Appropriate foliar application of plant growth regulators can mitigate heat-induced flag leaf senescence, prolong leaf green retention, and improve photosynthetic efficiency, thereby sustaining high photosynthetic capacity in heat-stressed wheat (Yaakob et al., 2021; Li et al., 2023a, 2023). Collectively, the ameliorative effects of EBR, MT, and Mix against heat injury in wheat stem from coordinated improvements in chlorophyll content, leaf functional duration, photosynthetic leaf area, and photosynthetic performance. These observations align with earlier reports concerning stress regulation by exogenous growth regulators in crops (Nie et al., 2015; Alam et al., 2018; Abdelrahman et al., 2020; Zhang et al., 2023).

### 4.3 Regulatory effects of exogenous plant growth regulator application on the reactive oxygen species scavenging mechanism of wheat under natural high temperature stress

Excessive accumulation of intracellular reactive oxygen species (ROS), including superoxide anion, hydrogen peroxide, and malondialdehyde (MDA), constitutes a core mechanism responsible for heat-triggered cellular injury in plants. These toxic molecules initiate membrane lipid peroxidation, compromise cell membrane integrity, and ultimately provoke severe oxidative stress (Martineau et al., 1979; Zhang and Sharkey, 2009; Wang et al., 2010; You and Chan, 2015; Zhou et al., 2019; Li et al., 2022; Mansoor et al., 2022). To resist adverse stresses, plants possess a sophisticated antioxidant defense system including enzymatic and non-enzymatic antioxidants. The enzymatic components encompass superoxide dismutase (SOD), peroxidase (POD), catalase (CAT), and glutathione reductase (GR), whereas glutathione (GSH) and ascorbic acid (AsA) represent the major non-enzymatic antioxidants (Zhou et al., 2019; Zhang et al., 2023). Modulation of antioxidant homeostasis is widely acknowledged as a key mechanism by which exogenous plant growth regulators mitigate heat-induced oxidative injury in crops (Kahraman et al., 2019). In this study, natural high temperature stress after anthesis disrupted the dynamic balance between ROS/MDA production and scavenging in wheat flag leaves, causing massive ROS and MDA accumulation and aggravated membrane oxidative damage. Nevertheless, the combined Mix treatment markedly reduced MDA and H_2_O_2_ concentrations and suppressed O_2_^-^ production, while substantially boosting endogenous GSH and AsA pools. Furthermore, the activities of key antioxidant enzymes (SOD, POD, CAT, GPX and APX) were markedly increased under the Mix treatment. These findings indicate that the combined application of optimized plant growth regulators effectively suppresses excessive ROS burst, enhances both enzymatic and non-enzymatic antioxidant capacity in wheat flag leaves, and alleviates ROS-mediated oxidative damage under heat stress. Moreover, the ameliorative effect of the Mix treatment was greater than that of individual foliar regulators, which can be attributed to the combined actions of multiple exogenous spray reagents. These results are consistent with previous work showing that foliar exogenous regulators and fertilizers enhance plant thermotolerance (Xue et al., 2012; Fahad et al., 2016; Lv et al., 2017; Gupta et al., 2018; Yang et al., 2022; Li et al., 2023a, 2023). Collectively, rational combined application of plant growth regulators can increase antioxidant enzyme activities and non-enzymatic antioxidant concentrations in wheat leaves, mitigate heat-triggered cellular oxidative damage, and represents an effective strategy to reduce the adverse effects of natural high temperature stress after anthesis on wheat productivity.

### 4.4 Regulatory effects of exogenous plant growth regulator application on transcriptional response mechanisms of wheat under natural high temperature stress

To mitigate heat-induced cellular injury, plants have evolved multilayered physiological, biochemical, and transcriptional regulatory strategies to cope with stress damage (Alam et al., 2018). Transcriptome sequencing has become an indispensable and powerful approach for exploring plant abiotic stress tolerance mechanisms, dissecting complex signal transduction cascades, and identifying core functional stress-responsive genes (Dinneny et al., 2008; Todd et al., 2016). Given that winter wheat is highly susceptible to natural high temperature stress after anthesis, this study hypothesized that foliar application of EBR and MT could trigger transcriptional reprogramming of heat-responsive genes in wheat flag leaves, thereby improving crop thermotolerance. Transcriptomic results demonstrated that EBR and MT treatments significantly activated carbohydrate metabolism pathways, including photosynthesis, the Calvin cycle, and general carbon metabolism. Under stress conditions, plants activate stress-responsive genes and modulate signaling networks to synthesize functional proteins, establishing integrated physiological and metabolic adaptations to hostile environments (Mittler et al., 2012; Mansoor et al., 2022). In contrast, severe heat stress disrupts cellular homeostasis and impairs antioxidant defense systems, causing excessive MDA accumulation, deteriorated membrane stability, and suppressed photosynthetic capacity (Zhang and Sharkey, 2009; Gururani et al., 2015; Li et al., 2022; Mansoor et al., 2022). Transcriptome profiling in this study identified a set of pivotal genes associated with photosynthesis and carbohydrate metabolism responsive to EBR and MT treatments, including ATPF, PET, PSB, GAPDH, PRK, ALDO, and FBP. The differential expression of these core genes represents a key molecular mechanism by which EBR and MT improve photosynthetic performance and heat tolerance in wheat flag leaves. These findings align with previous studies reporting that exogenous melatonin enhances thermotolerance in Arabidopsis and tall fescue (Shi et al., 2015; Alam et al., 2018). Collectively, these findings confirm that EBR and MT treatments facilitate the maintenance of photosynthetic machinery stability and mitigate heat-induced physiological damage in wheat. Furthermore, KEGG enrichment analysis showed that the glutathione metabolism pathway was significantly enriched in both EBR and MT comparison groups. Multiple key genes involved in glutathione metabolism and ROS scavenging (e.g., GPX, APX, GST, RRM, and GGT) exhibited distinct expression patterns under exogenous regulator treatments. Notably, the core antioxidant genes GST, GPX, and APX were markedly upregulated relative to the CK. The transcriptional changes in glutathione-mediated antioxidant metabolism substantially activate the defense response of wheat to natural high temperature stress after anthesis, thereby synergistically improving the molecular heat adaptability of winter wheat.

In summary, high-temperature stress occurring during the grain-filling period severely inhibits the growth and yield formation of winter wheat and poses a substantial threat to global food security. This study confirms that foliar application of exogenous plant growth regulators can effectively alleviate heat damage in wheat via the coordinated regulation of photosynthesis and antioxidant defense systems. A regulatory model illustrating how these regulators mediate wheat responses to natural high temperature stress after anthesis is presented in **Figure 10**.

**Figure 10:**
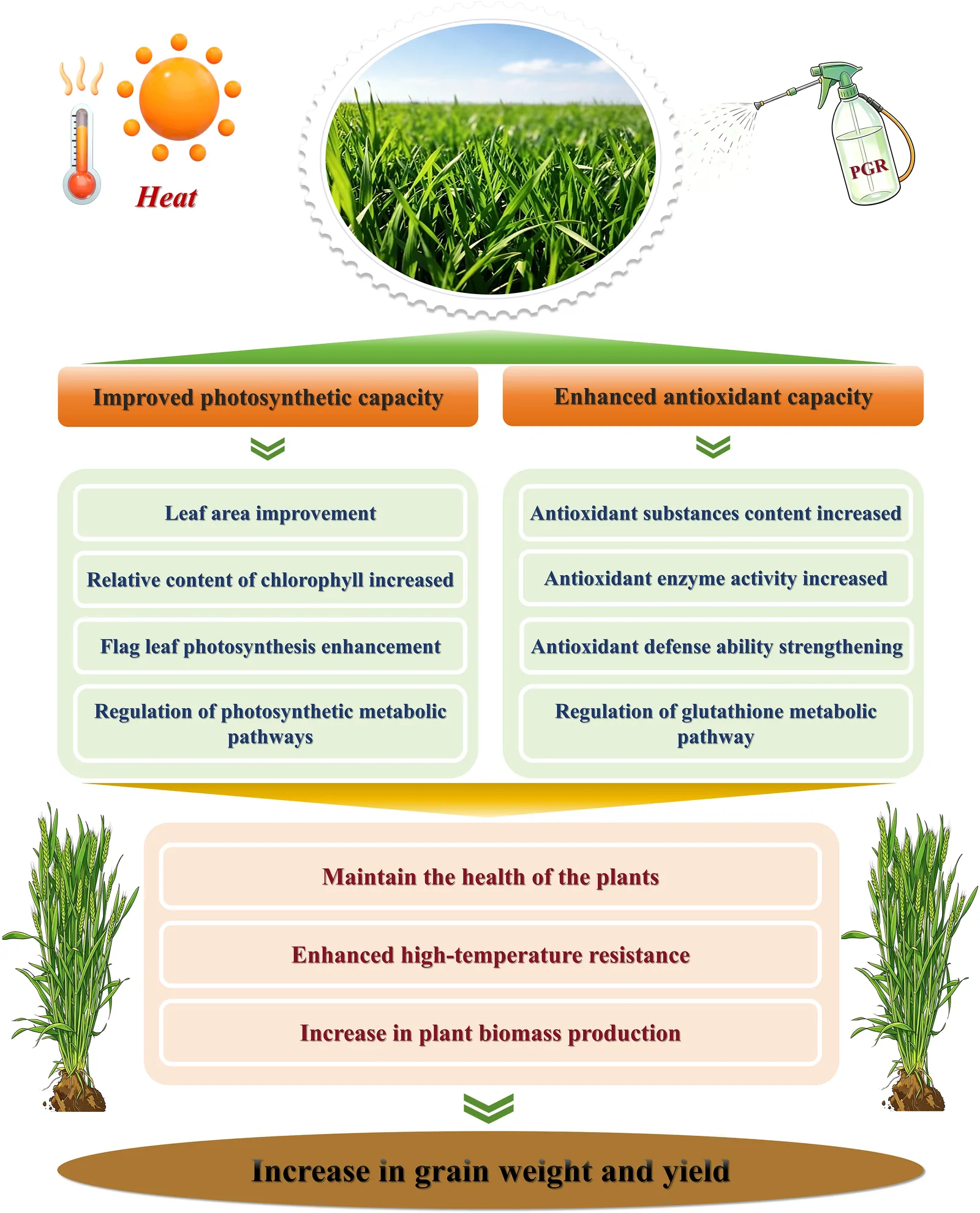
Regulatory mechanism model of exogenous plant growth regulators mediated wheat tolerance to natural high temperature stress after anthesis.

## 5 Conclusions

In the present study, exogenous plant growth regulators modulated the transcription of core photosynthetic-related genes, elevated the net photosynthetic rate of flag leaves, and consequently improved photosynthetic capacity to alleviate the damage caused by high temperature stress to wheat. Moreover, these exogenous regulators altered the transcript abundance of genes encoding key antioxidant enzymes, which activated antioxidant enzyme activities and relieved membrane lipid peroxidation. The synergistic regulation of photosynthetic and antioxidant metabolic pathways markedly enhanced the thermotolerance of winter wheat under natural high temperature stress after anthesis. Notably, the combined foliar treatment (0.05 µmol L^-1^ 2,4-epibrassinolide + 50 µmol L^-1^ melatonin + 15 mmol L^-1^ KH_2_PO_4_) substantially optimized the physiological-biochemical status of wheat plants, reduced biomass loss, and increased grain yield. Collectively, this composite regulator mixture can effectively strengthen the adaptability of winter wheat to natural high temperature stress at the grain-filling stage and holds promising potential for improving crop thermotolerance. This work elucidates the core regulatory mechanisms by which exogenous plant growth regulators mediate thermotolerance in winter wheat, and provides valuable theoretical references and practical implications for enhancing crop stress tolerance, facilitating wheat genetic improvement and optimizing field cultivation management strategies.

## Data Availability

The original contributions presented in the study are publicly available. The repository and corresponding BioProject accession number are listed as follows: NCBI (https://www.ncbi.nlm.nih.gov/), under accession number PRJNA1532874.
